# A third species of the rarely collected Oriental hoverfly genus *Furcantenna* Cheng, 2008 (Diptera, Syrphidae, Microdontinae)

**DOI:** 10.3897/zookeys.989.57087

**Published:** 2020-11-09

**Authors:** Menno Reemer

**Affiliations:** 1 Naturalis Biodiversity Center, P.O. Box 9517, 2300, RA Leiden, the Netherlands Naturalis Biodiversity Center Leiden Netherlands

**Keywords:** Identification key, Malaysia, morphology, new species, taxonomy

## Abstract

*Furcantenna malayana***sp. nov.** is described from Peninsular Malaysia, based on a single female collected in 1962. The other two known species of this genus are also known from single specimens, from Southeastern China and Nepal. A key to the species is given, and the taxonomy and distribution of the genus are discussed.

## Introduction

Few species of Syrphidae are known in which the basal antennal flagellomere (the apical segment of the antenna, which carries the arista) is furcate. In the subfamily Eristalinae a bifurcate basal antennal flagellomere is only found in males of the genus *Cacoceria* Hull, 1936. All other instances of this remarkable character among Syrphidae are restricted to the subfamily Microdontinae. In this subfamily, the character is known in the New World genera *Carreramyia* Doesburg, 1966, *Masarygus* Brèthes, 1909, *Schizoceratomyia* Carrera, Lopes & Lane, 1947 and an unnamed genus; in the Australian genus *Cervicorniphora* Hull, 1945; and in the Oriental genus *Furcantenna* Cheng, 2008 (Reemer and Ståhls 2013a).

The genus *Furcantenna* was described based on a single male specimen of *F. yangi* Cheng, 2008, collected in 1982 in the Guangxi Province in the Oriental part of China (Cheng and Thompson 2008). The second species, *F. nepalensis* Reemer, 2013, was described based on a single male specimen collected in 1967 in Nepal (Reemer and Ståhls 2013a). Ongoing taxonomic research in various entomological collections by the author revealed a third specimen of the genus, belonging to a third species, collected in 1962 in Peninsular Malaysia. This species is described here, and a key to distinguish it from the other two *Furcantenna* species is given.

## Material and methods

The studied specimen is deposited in the collection of the California Academy of Sciences (CAS) in San Francisco, USA. Photos of the specimen were made through a Zeiss Stereo Discovery v12 microscope, and processed (focus stacking) by Axiovision (version SE64 4.9) software. The map was made in Adobe Illustrator, based on locality information in Cheng and Thompson (2008), Reemer and Ståhls (2013a) and the present article (the symbols for the localities were placed by approximation, as available locality information is imprecise). Measurements of the studied specimen were taken using an ocular micrometer in a Wild M3B stereo microscope. Body length was measured from the anterior part of head (excluding antenna) to the apex of the abdomen. Morphological terminology follows Cumming and Wood (2017). For the type specimen, label data are given in quotation marks (“...”) and line breaks on the label are indicated with a slash (/).

## Results

### Generic diagnosis

The genus *Furcantenna* is characterized by the combination of the following characters: wing vein R_4+5_ without posterior appendix; wing vein M_1_ straight, perpendicular to wing vein R_4+5_; abdomen broadly oval; antenna inserted below dorsal eye margin; basal antennal flagellomere much longer than scape, longer than distance between antennal socket and anterior oral margin; scutellum apicomedially sulcate; metasternum developed.

The pilosity of the katepisternum is variable: bare in *F. yangi* (Cheng and Thompson 2008), pilose in the other two species. The pilosity of the metasternum is also variable: bare in *F. malayana* sp. nov., pilose in the other two species.

### Key to the species of *Furcantenna*

**Table:** 

1	Face and vertex black. Wing in apical half dark, at least anteriorly. Metasternum pilose. Katepimeron pilose or bare	**2**
–	Face medially and vertex partly yellow (Figs 1–4). Wing in apical half whitish (Fig. 7). Metasternum bare. Katepimeron pilose	***F. malayana* Reemer, sp. nov.**
2	Body colour brownish without violet shine. Katepimeron pilose. Scutellum slightly sulcate	***F. nepalensis* Reemer**
–	Body colour blackish with violet shine. Katepimeron bare. Scutellum deeply sulcate	***F. yangi* Cheng**

#### Furcantenna malayana sp. nov.

Taxon classificationAnimaliaDipteraSyrphidae

47DCE6A6-1A17-5C8C-9F3D-EA1D6D4F402A

http://zoobank.org/DED6DB6F-ADDA-4027-A0CB-295956C9CC47

Figures 1–8

##### Material.

***Holotype***: Malaysia. • 1 ♀, holotype of *Furcantenna malayana* sp. nov.; SE of Ipoh; alt. 50 m; 10 Oct. 1962; E.S. Ross & D.Q. Cavagnaro leg.(CAS).

Label 1: “MALAYA: 3 mi. / SE. Ipoh / 50m VII-10-62”; label 2: “Collectors: / E.S. Ross / D.Q. Cavagnaro”.

##### Diagnosis.

*Furcantenna malayana* is the only known species of the genus with a partly yellow face and vertex, and a bare metasternum. The whitish apical half of the wing is also characteristic.

##### Description.

**Figures 1–8.**
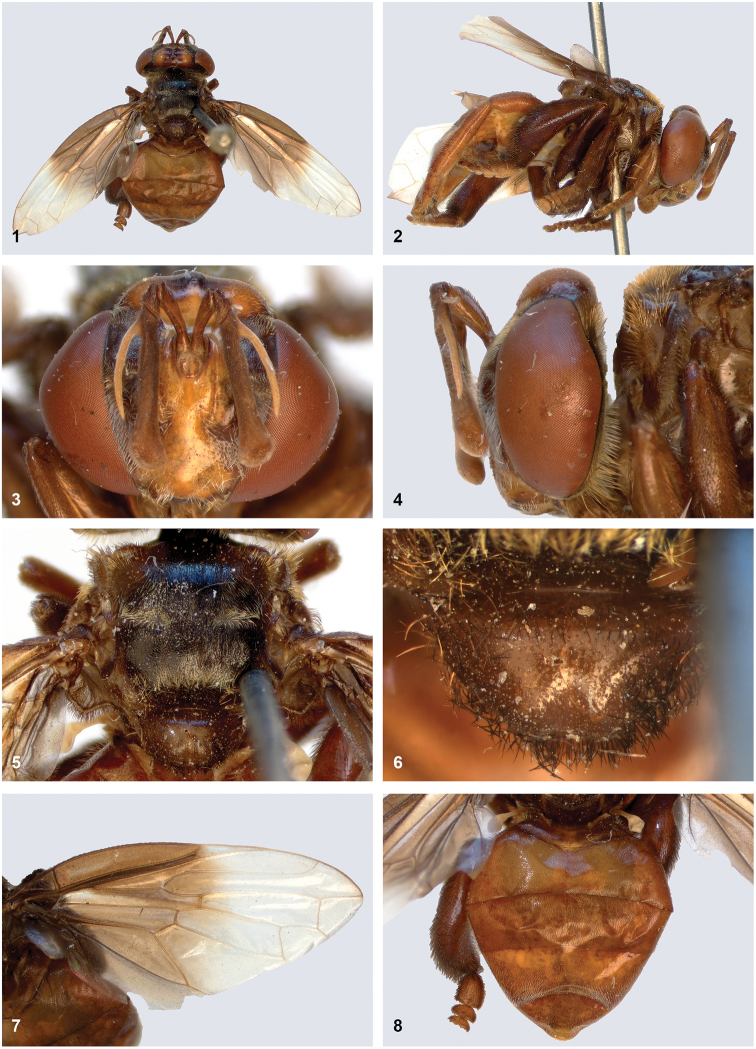
*Furcantenna malayana* sp. nov.,female, holotype **1** habitus dorsal **2** habitus lateral **3** head frontal **4** head lateral **5** thorax dorsal **6** scutellum dorsal **7** wing **8** abdomen dorsal.

**Adult female.** Body length: 9 mm (Fig. 1).

***Head*.** Face occupying slightly more than 1/2 of head width in frontal view; yellow medially, dark brown laterally; silvery white pilose. Oral cavity with lateral margins not produced. Frons medially yellow, bare; laterally dark brown, golden yellow pilose. Vertex swollen; yellow, except brown around ocellar triangle; yellow pilose. Occiput dark brown; yellow pilose. Eye bare, except for a few scattered, very short pile, only visible under high magnification (Fig. 4). Antennal socket about 1.5× as high as wide. Antenna brown; ratio of scape:basal flagellomere approx. 1:2.1; basal flagellomere swollen at apical 1/4, appearing club-shaped, with apex approx. twice as wide as base. Arista yellowish, about 3/4 as long as basal flagellomere (Fig. 3).

***Thorax*.** Postpronotum yellowish brown; yellow pilose. Mesoscutum brown, quite pale along margins and more blackish in middle; golden yellow pilose in anterolateral corners and along posterior margin, leaving a black pilose area in the shape of an upside-down letter T in between (Fig. 5). Postalar callus yellowish brown; yellow pilose. Scutellum without calcars, with shallow apicomedian sulcus; pale brown; black pilose (Fig. 6). Pleuron brown. Anterior and posterior part of anepisternum divided by a weak sulcus; almost entirely yellow pilose, except bare on narrow strip along dividing sulcus and on small ventral part. Anepimeron on anterior part with mixed yellow and black pile, other parts bare. Katatergum and anatergum long and short microtrichose, respectively. Katepisternum dorsally whitish pilose, ventrally bare. Katepimeron white pilose. Metasternum bare (except for dark microtrichia). Mediotergite dark brown; shining, except narrowly microtrichose anteriorly and on rudimentary subscutellum. Calypter grey. Halter pale yellow.

***Wing*.** Brown in basal half, whitish in apical half (Fig. 7). Microtrichose, except bare on posterobasal 1/2 of cell br.

***Legs*.** Pale brown, except hind femur and tibia darker brown; black pilose, except ventral surface of tarsi yellow pilose. Hind tibia strongly swollen, widest in the middle, with dorsal pile much longer than on other parts of the legs (Fig. 2). Coxae and trochanters brown; black pilose.

***Abdomen*.** Pale brown (Fig. 8). Tergites short pilose: tergite 1 yellow pilose; tergite 2 black pilose except yellow pilose along anterior margin; tergite 3 black pilose; tergites 4 and 5 yellow pilose. All sternites yellow pilose.

##### Etymology.

The specific epithet is an adjective which refers to Malaysia, the country where the species was collected.

##### Distribution.

**Figure 9.**
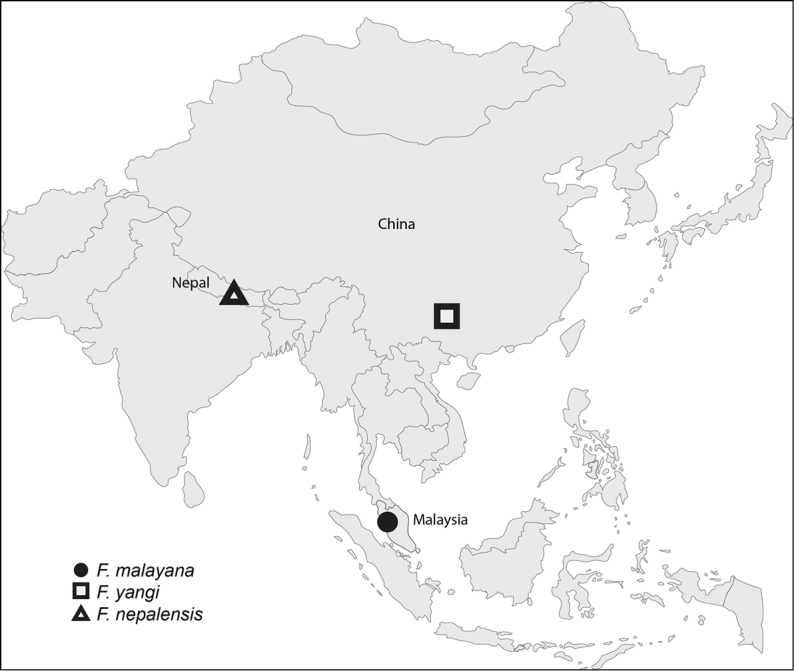
Known localities of *Furcantenna* species.

Only known from Peninsular Malaysia (Fig. 9).

## Discussion

The description of *Furcantenna malayana* is based on the first known female of the genus. Even though the basal antennal flagellomere is not furcate, as it is in the males of the other two described species of *Furcantenna*, the specimen is considered to belong to this genus. The combination of the following characters supports this placement: wing vein R_4+5_ without posterior appendix; wing vein M_1_ straight, perpendicular to wing vein R_4+5_; abdomen broadly oval; basal antennal flagellomere longer than distance between antennal socket and anterior oral margin; scutellum apicomedially sulcate; katepisternum pilose. Also, in most Neotropical counterparts of *Furcantenna* (species of *Carreramyia*, *Masarygus* and *Schizoceratomyia*) the furcate antenna is found only in males, with the exception of *Schizoceratomyia malleri* (Curran, 1947) and *Masarygus carrerai* Papavero, 1962 (the female of the Australian genus *Cervicorniphora* is unknown).

*Furcantenna* was placed in a clade with the Neotropical genera *Carreramyia*, *Masarygus*, and *Schizoceratomyia* in a phylogenetic analysis based on morphological characters (Reemer and Ståhls 2013b). If these genera are indeed closely related, this would be a case of a trans-Pacific distribution pattern as defined by Cranston (2005). Similar examples among Microdontinae are found in the subgenus Chymophila Macquart, 1834 of *Microdon* Meigen, 1803, and in the genus *Paramicrodon* de Meijere, 1913 (Reemer 2012).

Apparently the genus *Furcantenna* is quite widespread in the Oriental region, even though the species are very rarely collected. Whether their elusiveness is a result of very restricted ranges, occurrence in very low densities, or a lifestyle that makes them difficult to notice, remains a matter of speculation.

## Supplementary Material

XML Treatment for Furcantenna malayana
